# Goal Attainment Among Older Adults With Ischemic Heart Disease Using Mobile-Health Cardiac Rehabilitation in RESILIENT

**DOI:** 10.1016/j.jacadv.2025.102298

**Published:** 2025-11-12

**Authors:** Elianna M. Shwayder, John A. Dodson, Samrachana Adhikari, Eleonore V. Grant, Antoinette M. Schoenthaler, Stephanie Pena, Yuchen Meng, Lee A. Jennings

**Affiliations:** a Brigham and Women’s Hospital, Boston, Massachusetts, USA; b New York University Langone Medical Center, New York, New York, USA; c Yale School of Medicine, New Haven, Connecticut, USA; d The University of Oklahoma Health Sciences Center, Section of Geriatrics and Palliative Medicine, Oklahoma City, USA

**Keywords:** aging, mobile-health cardiac rehabilitation, patient-centered outcomes

## Abstract

**Background:**

Data on patient-centered outcomes of mobile health cardiac rehabilitation (mHealth-CR) for older adults with ischemic heart disease are limited. The RESILIENT (Rehabilitation at Home Using Mobile Health in Older Adults After Hospitalization for Ischemic Heart Disease) trial, the largest randomized study of mHealth-CR in this population, found no significant improvements in functional capacity, health status, angina, or disability compared with usual care.

**Objectives:**

The purpose of this study was to evaluate whether mHealth-CR affects personalized goal attainment—a prespecified secondary endpoint of RESILIENT—using goal attainment scaling (GAS).

**Methods:**

A total of 400 patients (≥65 years) with ischemic heart disease were randomized to mHealth-CR or usual care. Participants specified goals for CR at baseline using the five-category goal attainment scale: much-less-than-expected (−2), less-than-expected (−1), expected (0), better-than-expected (+1), and much-better-than-expected (+2). Goal attainment was assessed at 3 months.

**Results:**

Of 400 patients (median age, 71.0 years [range 65.0-91.0]; 72.8% male; 65.2% prefrail/frail) randomized to mHealth-CR (n = 298) or usual care (n = 102), 353 (88.3%) completed GAS. Most goals addressed physical activity (54.0% mHealth-CR vs 59.0% usual care), health care behaviors (14.4% vs 11.9%), or symptom management (13.1% vs 9.0%). Rates of attaining or exceeding goals (GAS ≥0) were similar between groups (80.5% vs 77.6%; *P* = 0.492). However, in the intervention arm, there was a higher rate of exceeding expected level of goal attainment (GAS +1, +2) compared with usual care (52.6% vs 34.2%; *P* = 0.006).

**Conclusions:**

In a trial that did not demonstrate differences on traditional endpoints, those receiving mHealth-CR were more likely to exceed personalized CR goals. These findings suggest the intervention facilitated greater progress toward individualized goals and underscore the importance of patient-centered outcomes in CR. (Rehabilitation at Home Using Mobile Health In Older Adults After Hospitalization for Ischemic Heart Disease [RESILIENT]; NCT03978130)

Cardiac rehabilitation (CR) for patients with ischemic heart disease (IHD) is a cornerstone of secondary prevention, with decades of research demonstrating that CR reduces cardiovascular events and improves quality of life.^1^ However, participation among older adults remains disproportionately low, driven by barriers such as physical limitations, sensory impairments, transportation challenges, limited social support, and inadequate physician referral.^1^, ^2^, ^3^, ^4^, ^5^, ^6^, ^7^, ^8^

Mobile health CR (mHealth-CR) has emerged as a potential strategy to address these barriers by offering a more accessible alternative to traditional center-based programs. Despite its promise, there is limited evidence supporting the efficacy of mHealth-CR in older adults.^1^^,^^6^^,^^9^^,^^10^ In this context, the RESILIENT (Rehabilitation at Home Using Mobile Health in Older Adults After Hospitalization for Ischemic Heart Disease) trial^11^^,^^12^ was a pragmatic randomized clinical trial that was designed to test whether mHealth-CR improved functional capacity (6-minute walk distance [6MWD]) and secondary outcomes (quality of life, residual angina, disability) compared with usual care. In RESILIENT, while mHealth-CR modestly increased 6MWD compared with usual care, this did not reach the threshold for statistical or clinical significance (≥25 m).^11^ Furthermore, secondary outcomes including health status, residual angina, and disability were similar between groups.

In addition to the metrics of functional capacity, health status, residual angina, and disability, the RESILIENT trial included goal attainment as a secondary outcome. Goal attainment scaling (GAS) is a method for evaluating the effect of an intervention on achievement of participant-defined goals and is particularly relevant to older adult populations with multiple comorbidities and varying functional capacities.^13^, ^14^, ^15^ While widely applied in geriatric and neurological rehabilitation, as well as in studies of rare diseases with heterogeneous symptoms, to date GAS has seldom been applied in the setting of CR.^16^, ^17^, ^18^, ^19^ Our study aimed to evaluate the effect of mHealth-CR on goal attainment compared with usual care.

## Methods

The RESILIENT trial (NCT03978130) evaluated whether mHealth-CR improved functional capacity as measured by 6MWD, the primary outcome, in older adults (age ≥65 years) with IHD compared with usual care, and has been described previously.^11^ In brief, participants were identified at the time of acute myocardial infarction (AMI), percutaneous coronary intervention (PCI), or coronary artery bypass graft (CABG). A total of 400 patients were randomized in a 3:1 ratio to mHealth-CR vs usual care. The mHealth-CR intervention included a personalized exercise program combining aerobic activity with low-level isotonic resistance training, supported by a software platform, weekly counseling sessions with an exercise therapist, and remote physiological monitoring via a Fitbit and blood pressure cuff. All exercise therapists held at least a Master’s degree in Exercise Science. RESILIENT included the following elements consistent with a pragmatic trial: 1) allowing participants in both study arms to still attend traditional CR at their cardiologist’s discretion; 2) delivery of the study intervention by currently employed clinical exercise therapists; 3) minimal exclusion criteria to better represent patients who would receive the intervention in standard practice; and 4) inclusion of multiple patient-reported outcome measures. Secondary outcomes for RESILIENT included goal attainment, health status (12-item short form health survey), basic and instrumental activities of daily living (Katz ADL^20^ and Lawton IADL^21^ scales), hospital readmissions, and mortality. All outcomes except for hospital readmission and goal attainment have been previously reported.^11^ The RESILIENT trial was approved by the New York University Grossman School of Medicine Institutional Review Board. We adhered to the Consolidated Standards of Reporting Trials guidelines in our analyses and manuscript preparation (Supplemental Table 3). This study is a secondary outcome analysis assessing goal attainment in the 400 patients with AMI, PCI, or CABG who enrolled in the RESILIENT trial and completed GAS at the time of CR enrollment.

### Goal attainment scaling

At baseline, participants were introduced to example goals across domains, such as health-related behaviors, physical activity, and social interaction, to facilitate discussion of their CR goals for the upcoming 3 months.^22^^,^^23^ Using the SMART (specific, measurable, achievable, relevant, and timely) goal framework, each participant set a single, individualized goal prior to randomization.^22^ A clinical research coordinator (CRC) led the initial goal-setting discussion and guided participants to refine and specify their goal using a 5-category scale to facilitate objective measurement at the 3-month endpoint: much-less-than-expected (−2), less-than-expected (−1), expected attainment (0), better-than-expected (+1), and much-better-than-expected (+2).^13^^,^^14^ Each goal attainment scale was examined for clinical relevance, objectivity, precision, and clarity, with baseline performance clearly identified.^24^ Participants also identified perceived barriers to achieving their goals and formulated action plans to address these obstacles. CRCs were trained by a GAS expert (L.A.J.) using a structured protocol that included didactic instruction, observed practice, and real-time feedback with example goals tailored to the study population. To support fidelity, a follow-up session after trial launch included review and discussion of GAS documentation from each CRC, clarification of questions, and additional guidance to reinforce standardized application of the GAS process. Examples that illustrate the process of GAS are included in Supplemental Table 1, and the data collection tools can be found in Supplemental Figure 1.

At 3 months, participants rated their level of goal attainment from −2 to +2 with support from a trained research assistant who was blinded to their treatment assignment, as part of the outcomes assessment for the trial. Each participant’s GAS score was transformed into a single T-score, allowing a standardized comparison using a simplified formula for one-goal GAS, as each participant set only one goal: *GAS = 50 + (10x)*, where x is the numerical value (−2 to +2) of the attainment level.^25^ A T-score of 50 reflects expected goal attainment.^14^

### Covariates and subgroups

Prognostic covariates—study site, age, diabetes status, and baseline 6MWD—were identified a priori given their plausible relationship with the primary study outcome. These covariates were adjusted for in the outcome analysis to enhance precision in effect estimation, in a similar manner to the primary endpoint analysis published previously.^11^ To investigate whether goal attainment varied by clinically relevant subgroups, we analyzed the relationship between GAS T-score and mHealth-CR across prespecified subgroups as an exploratory analysis: age, sex, body mass index, frailty, functional impairment, depression, enrollment criteria (AMI, elective PCI, CABG), diabetes, heart failure, chronic kidney disease, osteoarthritis, living alone, baseline 6MWD, as detailed in the primary endpoint analysis.^11^ We also examined whether participation in traditional CR, defined by self-reported attendance at ≥1 center-based session, influenced treatment effect. Frailty was assessed using the Fried frailty index, adapted for use with the Telephone Assessment of Physical Activity, and categorized as frail, prefrail, or robust.^26^ Functional impairment was defined as a score below 6 on the Katz Basic ADL scale or below 8 on the Lawton IADL scale.

### Statistical analysis

The ordinal GAS scores (−2 to +2) were summarized using the median, whereas the continuous GAS T scores were summarized using mean and 95% CIs. The distribution of ordinal GAS scores across the 2 treatment groups (prespecified secondary outcome) was assessed using a Wilcoxon-Mann-Whitney rank sum test.^27^ The Cochran-Armitage test was applied to evaluate whether a linear trend existed across ordered goal attainment categories between treatment groups. The mean difference in GAS T-score by arm was assessed using a linear regression model, adjusting for the prespecified covariates. To assess assumptions of the GAS T-score linear regression model, we evaluated variance inflation factors for multicollinearity, Q-Q plots for normality of residuals, and residual-versus-fitted plots for homoscedasticity. As an exploratory analysis, we dichotomized GAS scores (0, +1, +2 vs −1, −2) to evaluate the percentage of participants in each group who attained their goals using a chi-squared test. The outcome of GAS was analyzed using an intent-to-treat approach, based on the patients' initial randomization to the treatment arm. Therefore, analyses included all randomized participants including dropouts (intention-to-treat sample). Multiple imputation using chained equations was used to impute endpoints for individuals who did not complete the trial; estimates across the imputed data sets were pooled using Rubin’s rule as previously discussed in the primary paper.^11^^,^^28^ The proportion of missing values imputed for each outcome is presented in Supplemental Table 2. All statistical analyses were conducted using RStudio (Version 4.2.2).

### Analysis for heterogeneity of treatment effect

To assess whether the treatment effect for the continuous GAS T-scores differed across subgroups, interaction terms between treatment and subgroup variables were estimated in linear regression models. No adjustments were made for multiplicity, and CIs for the group-specific effects should be interpreted cautiously, as these subgroup analyses are exploratory.

### Qualitative analysis

As previously described,^29^ a random sample of 100 goals specified at baseline was analyzed using a combination of deductive (a priori) and inductive (emergent) coding approaches.^30^ The initial coding framework of participants’ goals was derived from the 4 domains of the Patient Priorities Care Values Framework—managing health, enjoying life, improving function, and connecting with others—a model designed to align health care delivery with the values of older adults with multiple comorbidities.^23^ Through inductive coding, additional granular subdomains emerged, reflecting the diversity of participant-defined goals. In this study, we applied this framework^29^ to the remaining 300 baseline goals which were coded by 2 independent reviewers (E.M.S., E.V.G.). Discrepancies were resolved by group consensus among study investigators to ensure consistency.

### Exploratory analyses

To examine whether individual-level changes in physical function were associated with goal attainment, we regressed GAS T-score on change in 6MWD, adjusting for the aforementioned prespecified covariates. We also explored how goal type (dichotomized to physical activity goal vs not related to physical activity) was distributed across prespecified clinical subgroups^11^ and treatment arm using chi-squared tests. The subgroups were selected post hoc if their subgroup-specific mean GAS T-score difference exceeded the overall mean GAS T-score difference between treatment arms.

## Results

### Patients

Baseline characteristics of the 400 participants enrolled in the RESILIENT trial have been previously described and are presented in Table 1.^11^ Of these participants, there were 353 with goal attainment scores at 3 months. Missing endpoint data from 29 participants in the mHealth-CR group and 18 in the usual care group were subsequently imputed in the outcome analysis. Briefly, the cohort had a median age of 71.0 years (range: 65.0-91.0 years) with 105 (26.2%) participants age 75 or older. Most participants were enrolled based on the criteria of elective PCI (N = 254, 63.5%). A majority of participants were classified as frail or prefrail (N = 261, 65.2%), and 73 (18.2%) reported at least one impairment in ADLs or IADLs. In the intervention arm, 38 (12.8%) attended traditional center-based CR, compared with 26 (25.5%) in the usual care arm.

**Table 1:** RESILIENT Trial Demographic, Clinical Characteristics, and Goal Domains at Baseline (N = 400)

	mHealth-CR (n = 298)	Usual Care (n = 102)
Demographics		
Age, y	71 (65-91)	71 (65-89)
Male sex	216 (72.5%)	75 (73.5%)
Race		
Asian	11 (3.7%)	6 (5.9%)
Black	27 (9.1%)	9 (8.8%)
Multiple races or other^a^	31 (10.4%)	13 (12.7%)
White	229 (76.8%)	74 (72.5%)
Latinx or Hispanic ethnicity	23 (7.7%)	11 (10.8%)
Insurance status		
Medicare	228 (76.5%)	83 (81.4%)
Medicaid	33 (11.1%)	11 (10.8%)
Private insurance	211 (70.8%)	73 (71.6%)
Clinical characteristics		
Enrollment criteria		
Acute coronary syndrome		
Acute myocardial infarction with PCI	74 (24.8%)	22 (21.6%)
Acute myocardial infarction without PCI	7 (2.3%)	2 (2.0%)
Unstable angina with PCI	18 (6.0%)	5 (4.9%)
Elective PCI	186 (62.4%)	68 (66.7%)
Coronary artery bypass graft	13 (4.4%)	5 (4.9%)
BMI, median (range)	27.3 (14.7-46.8)	27.7 (18.4-44.4)
Hypertension	254 (85.2%)	83 (81.4%)
Heart failure	34 (11.4%)	13 (12.7%)
Diabetes mellitus	94 (31.5%)	42 (41.2%)
Chronic lung disease	40 (13.4%)	13 (12.7%)
eGFR, mL/min/1.73 m^2^		
Mean (SD)	76.9 (24.2)	75.3 (16.7)
eGFR <60	47 (15.8%)	21 (20.6%)
Osteoarthritis	54 (18.1%)	24 (23.5%)
History of any tobacco use	75 (25.2%)	22 (21.6%)
ADL or IADL impairment	54 (18.1%)	19 (18.6%)
Frailty^b^		
Frail	37 (12.4%)	6 (5.9%)
Prefrail	154 (51.7%)	64 (62.7%)
Robust	82 (27.5%)	25 (24.5%)
Depressive symptoms^c^	35 (11.7%)	10 (9.8%)
Goal domains		
Physical activity	210 (54.0%)	79 (59.0%)
Symptom management	51 (13.1%)	12 (9.0%)
Health care or health-related behaviors	56 (14.4%)	16 (11.9%)
Enjoying life	46 (11.8%)	15 (11.2%)
Connecting with others	17 (4.4%)	9 (6.7%)
Improving function	9 (2.3%)	3 (2.2%)

Values are median (Q1-Q3) or n (%).

ADL = activity of daily living; BMI = body mass index; eGFR = estimated glomerular filtration rate; IADL = instrumental activity of daily living; mHealth-CR = mobile health cardiac rehabilitation; PCI = percutaneous coronary intervention; RESILIENT = Rehabilitation at Home Using Mobile Health in Older Adults After Hospitalization for Ischemic Heart Disease.

a Based on self-identified category. Other includes American Indian or Alaska Native, Native Hawaiian or other Pacific Islander, or participants self-selecting “other” without further specification.

b Defined using Fried et al modified by the Telephone Assessment of Physical Activity (TAPA).^26^^,^^31^

c Based on the 8-item Patient Health Questionnaire.^32^

### Goal content

Goal types were similarly distributed between treatment arms (Table 1). The majority of goals focused on physical activity (54.0% mHealth-CR vs 59.0% usual care), typically involving walking or combining walking with additional activities. Goals were also commonly related to health care or health-related behaviors (14.4% vs 11.9%), such as weight loss, preventing hospitalization, dietary changes, or smoking cessation. Goals targeting blood pressure or cholesterol management were rare (0.6% vs 0.0%). Symptom management was another prevalent focus (13.1% vs 9.0%), with participants wanting to reduce the burden of shortness of breath, fatigue, or chest pain. Participants also prioritized enhancing enjoyment of life (11.8% vs 11.2%) through productivity or recreational activities. Few goals related to social connection (4.4% vs 6.7%) or improving function (2.3% vs 2.2%).

### Goal attainment

#### Goal attainment in mHealth-CR vs usual care

Goal attainment (GAS 0, +1, +2 vs −1, −2) was similar between groups (mHealth-CR 80.5% vs usual care 77.6%; *P* = 0.492). However, in the mHealth-CR arm, there was a higher rate of exceeding expected level of goal attainment (GAS +1, +2) compared with usual care (52.6% vs 34.2%; *P* = 0.006), and a lower rate of only meeting expected level of goal attainment (GAS 0: 27.9% vs 43.4%; *P* = 0.006). The Cochran-Armitage test demonstrated a significant linear trend across ordered goal attainment categories (−2 to +2) between randomization groups (*P* = 0.013). Mean GAS T-scores with covariate adjustment demonstrated higher T-scores in the mHealth-CR group compared to usual care (54.8 [95% CI: 53.4-56.2] vs 51.2 [95% CI: 48.8-53.6], mean difference = 3.6 [95% CI: 0.8-6.4]; *P* = 0.012) (Table 2). For the GAS T-score linear regression model, variance inflation factors near 1 indicated no multicollinearity, Q-Q plots showed residuals were approximately normally distributed, and residual-versus-fitted plots confirmed homoscedasticity.

**Table 2:** Patient Goal Attainment at 3 Months

Outcome^a^	mHealth-CR		Usual Care		*P* Value
Patient goal attainment					0.006^b^
+2 Much better than expected	63 (25.5%)	130 (52.6%)	13 (17.1%)	26 (34.2%)	
+1 Somewhat better than expected	67 (27.1%)		13 (17.1%)		
0 Expected level of goal attainment	69 (27.9%)	69 (27.9%)	33 (43.4%)	33 (43.4%)	
−1 Somewhat less than expected	31 (12.6%)	48 (19.5%)	10 (13.2%)	17 (22.4%)	
−2 Much less than expected	17 (6.9%)		7 (9.2%)		
Mean GAS T-score (95% CI)	54.8 (53.4-56.2)		51.2 (48.8-53.6)		0.012^c^

GAS = goal attainment scaling; other abbreviation as in Table 1.

Across ordered goal attainment categories (−2 to +2), the Cochran-Armitage test demonstrated a significant linear trend between randomization groups (*P* = 0.013).

a For outcome comparisons, missing GAS scores at 3 months were imputed as described in the Methods.

b Wilcoxon-Mann-Whitney rank sum test.

c T-test for comparison.

#### Analysis for heterogeneity of treatment effect

**Figure 1:**
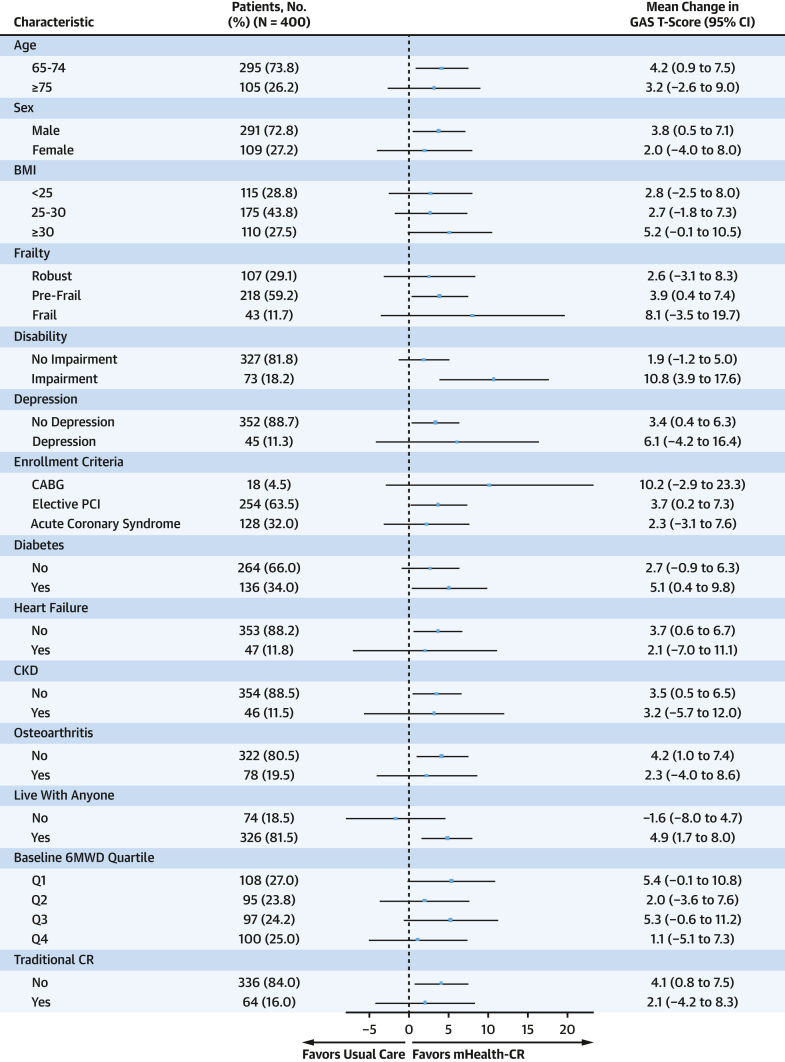
**Effect of the Study Intervention on Goal Attainment by Subgroup Forest Plot Showing the Treatment Effect of mHealth-CR vs Usual Care on Goal Attainment Across Prespecified Subgroups** Thirty-two patients had missing frailty data, and three had missing depression data. BMI = body mass index; CABG = coronary artery bypass graft; CKD = chronic kidney disease; CR = cardiac rehabilitation; GAS = goal attainment scaling; mHealth-CR = mobile health cardiac rehabilitation; PCI = percutaneous coronary intervention; 6MWD = six-minute walk distance.

The effect of mHealth-CR on goal attainment across prespecified subgroups is summarized in Figure 1. Subgroups in which the adjusted GAS T-score difference between mHealth-CR and usual care exceeded the overall mean difference (3.6 [95% CI: 0.8-6.4]) included participants aged 65 to 74 years (4.2 [95% CI: 0.9-7.5]), males (3.8 [95% CI: 0.5-7.1]), individuals who were prefrail (3.9 [95% CI: 0.4-7.4]), those with any ADL or IADL impairment (10.8 [95% CI: 3.9-17.6]), participants meeting enrollment criteria through elective PCI (3.7 [95% CI: 0.2-7.3]), individuals with diabetes (5.1 [95% CI: 0.4-9.8]), those without heart failure (3.7 [95% CI: 0.6-6.7]), participants without osteoarthritis (4.2 [95% CI: 1.0-7.4]), those living with others (4.9 [95% CI: 1.7-8.0]), and those who did not participate in traditional CR (4.1 [95% CI: 0.8-7.5]). Goal attainment favored usual care in only one subgroup: participants living alone (−1.6 [95% CI: −8.0 to 4.7]). A T-score change exceeding +10 reflects a shift to a different level of goal attainment on the 5-category response scale; this threshold was observed exclusively in participants in mHealth-CR with any ADL or IADL impairment (N = 54).

#### Exploratory outcomes

Change in 6MWD was not significantly associated with GAS T-score (β = 0.015; 95% CI: [-0.004-0.034]; *P* = 0.129). Chi-squared analyses revealed no significant differences in the frequency of physical activity-related goals compared with non-physical activity goals across treatment arm and subgroups.

## Discussion

Clinical trials in cardiovascular medicine have historically focused on disease-centered outcomes, placing less emphasis on individualized patient experiences and quality-of-life measures.^22^^,^^23^^,^^33^, ^34^, ^35^ More recently, while both the American Heart Association and the European Society of Cardiology have stressed the importance of individualized goal setting to improve patient activation and self-management, few trials in CR have integrated this patient-centered approach.^4^^,^^36^^,^^37^ To our knowledge, the present study is the first to systematically evaluate the individualized priorities of older adults with IHD before initiating CR and to investigate how an mHealth-CR program might facilitate the attainment of those personal goals. In this multicenter, pragmatic, randomized controlled trial, overall rates of goal achievement were similar between older adults receiving mHealth-CR vs usual care, as measured by GAS. However, participants in the mHealth-CR group were more likely to exceed their expected level of goal attainment (Central Illustration).

**Central Illustration:**
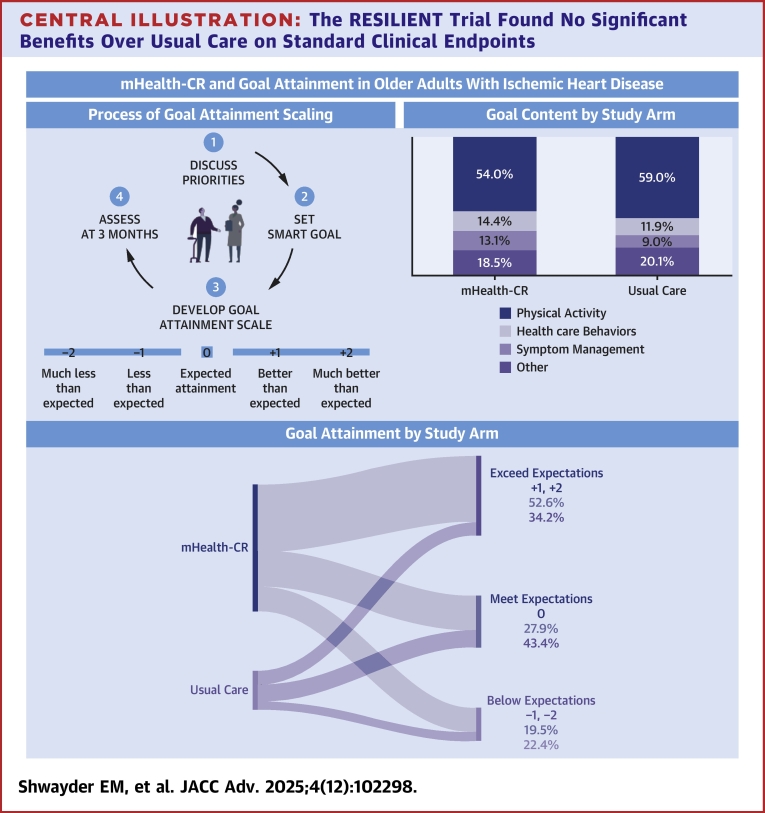
**The RESILIENT Trial Found No Significant Benefits Over Usual Care on Standard Clinical Endpoints** In this secondary analysis, participants completed goal attainment scaling (GAS) using a 5-point scale (−2 to +2) to establish individualized CR goals. Most goals addressed physical activity. While the overall proportion of participants achieving their expected goals (GAS ≥0) did not differ significantly between mHealth-CR and usual care (80.5% vs 77.6%; *P* = 0.492), mHealth-CR participants were more likely to exceed their expected level of goal attainment (+1, +2) compared with usual care (52.6% vs 34.2%, *P* = 0.006). Despite the absence of between-group differences on traditional clinical endpoints, a patient-centered measure revealed that mHealth-CR participants were more likely to exceed their goals for CR, highlighting the importance of incorporating patient-centered outcomes into CR programs and clinical trials. RESILIENT = Rehabilitation at Home Using Mobile Health in Older Adults After Hospitalization for Ischemic Heart Disease.

There are several explanations for our observed findings. Participants in mHealth-CR may have benefitted from structural components of the intervention—such as weekly check-ins with an exercise therapist and remote physiologic monitoring (FitBit and blood pressure cuff)—or the software platform itself. The majority of goals set by participants who received mHealth-CR were related to physical activity (54.0%), and in this context, meetings with the exercise therapist that were focused on reviewing perceived exertion and step count data may have prompted data-driven reflection and progress toward physical activity or quality-of-life goals. This highlights the potential for GAS to capture patient-centered outcomes that extend beyond disease-specific measures.

Goal attainment was notably enhanced among participants with self-reported baseline disability. Subgroup analyses revealed that among all subgroups, only individuals with ADL/IADL impairments in the mHealth-CR group achieved a mean T-score increase exceeding 10 (+10.8 [95% CI: 3.9-17.6]). This increase represents progression to one higher level of goal attainment, potentially signaling clinically meaningful improvement.^14^ Although participants with functional impairment receiving mHealth-CR improved their 6MWD beyond the clinically significant threshold of 25 m (mean change, 29.1 m [95% CI: −10.7 to 68.9 m]), this exploratory finding included the null hypothesis and should be interpreted with caution.^11^ In this context, GAS may serve as a more sensitive instrument to detect gains that are not captured by conventional instruments but are nonetheless meaningful to older adults. However, alternative explanations should also be considered, including lower baseline function that may have prompted conservative goal setting or enabled greater relative gains, as well as regression to the mean. Participants with functional impairment were as likely to set physical activity goals as participants without functional impairment, suggesting that their improvements were not attributable to the type of goal set. Our findings contrast with a recent trial in younger adults (mean age 60.1) enrolled in traditional CR, in which frail CR participants were less likely to achieve collaboratively set goals restricted to predefined categories (cardiovascular health, physical fitness, or weight loss).^18^ Collectively, these results highlight the personalized and nuanced nature of GAS and its potential to empower patients by accommodating individually relevant goals, which may more precisely capture multidimensional progress than standardized frameworks.^38^^,^^39^

### Study limitations

Our findings should be interpreted in the context of the study’s limitations. Participants may have overreported their success, introducing potential observer bias. Self-assessments may also be influenced by individual variation in optimism, self-efficacy, and mental health. Moreover, we did not find an association between GAS T-score and change in 6MWD. Thus, while GAS provides valuable insights into patient-centered outcomes, its subjective nature may limit generalizability. Future research should further investigate the application of GAS in mHealth-CR populations, especially as mobile technologies continue to evolve and gain traction among older adults. Understanding how individualized goal attainment relates to measurable clinical benefits will be critical for advancing patient-centered approaches in CR, particularly among older adults and underrepresented populations.

## Conclusions

Although no overall differences were observed in rates of meeting or exceeding expected goals, older adults receiving mHealth-CR—particularly those with functional impairments—were more likely to surpass their individualized CR goals. These findings underscore the potential of mHealth-CR to facilitate meaningful, patient-centered progress, even in the absence of differences in conventional clinical endpoints. Integrating tailored goal-setting frameworks that align with patients’ values and motivations can reveal the individualized benefits of CR. More broadly, these results highlight the importance of incorporating patient-centered outcomes into CR programs and clinical trials to optimize care and enhance impact for an increasingly diverse and aging IHD population.

Perspectives **COMPETENCY IN PATIENT CARE AND PROCEDURAL SKILLS:** Older adults are underengaged in traditional, center-based CR due to barriers such as physical and sensory impairments, transportation challenges, limited social support, and insufficient physician referrals. mHealth-CR has emerged as a valuable alternative to address these challenges. GAS, a sensitive and personalized outcome measure widely used in rehabilitation research, effectively captures meaningful progress across diverse functional abilities. This study demonstrates that while overall rates of goal attainment were similar between participants who received mHealth-CR as compared to usual care, participants who received mHealth-CR were more likely to exceed their expected level of goal attainment. This effect was particularly pronounced among participants with baseline impairments in ADL.

**TRANSLATIONAL OUTLOOK:** These findings highlight the importance of integrating patient-centered outcomes into CR programs and clinical trials to help optimize care for an increasingly aging population with IHD.

## Funding support and author disclosures

RESILIENT is funded by an 10.13039/100000002NIH/10.13039/100000049NIA grant (R01AG062520). Dr Dodson is supported by an 10.13039/100000002NIH/10.13039/100000049NIA mentoring award (K24AG080025). The sponsor had no role in the design, methods, data collection, analysis, or manuscript preparation. The authors have reported that they have no relationships relevant to the contents of this paper to disclose.
